# Integrated analysis of tumor-associated macrophage infiltration and prognosis in ovarian cancer

**DOI:** 10.18632/aging.203613

**Published:** 2021-10-11

**Authors:** Qianxia Tan, Huining Liu, Jie Xu, Yanqun Mo, Furong Dai

**Affiliations:** 1 Department of Gynecology and Obstetrics, Xiangya Hospital Central South University, Changsha, Hunan, China; 2 Department of Nephrology, Xiangya Hospital Central South University, Changsha, Hunan, China

**Keywords:** ovarian cancer, tumor immune infiltration, tumor-associated macrophages, WGCNA, prognosis

## Abstract

Ovarian cancer (OC) is a frequently lethal gynecologic malignancy, characterized by a poor prognosis and high recurrence rate. The immune microenvironment has been implicated in the progression of OC. We characterized the immune landscape in primary and malignant OC ascites using single-cell and bulk transcriptome raw OC data acquired from the Gene Expression Omnibus and The Cancer Genome Atlas databases. We then used the CIBERSORT deconvolution algorithm, weighted gene co-expression network analysis, univariate and multivariate Cox analyses, and the LASSO algorithm to develop a tumor-associated macrophage-related gene (TAMRG) prognostic signature, which enabled us to stratify and predict overall survival (OS) of OC patients. In addition, inter- and intra-patient heterogeneity of infiltrating immune cells was characterized at single-cell resolution. Tumor-infiltrating macrophages with an M2 phenotype exhibited immunosuppressive activity. M1 macrophages positively correlated with OS, whereas activated mast cells, neutrophils, M2 macrophages, and activated memory CD4^+^ T cells were all negatively correlated with OS. A total of 219 TAMRGs were identified, and a novel 6-gene signature (*TAP1*, *CD163*, *VSIG4*, *IGKV4-1*, *CD3E*, and *MS4A7*) with independent prognostic value was established. These results show that a TAMRG-based signature may be a promising prognostic and therapeutic target in OC.

## INTRODUCTION

With 13,770 estimated deaths in the United States in 2021, ovarian cancer (OC) has emerged as a highly lethal gynecologic malignancy [1]. Almost 80% of OC cases are diagnosed in advanced stages, and its 5-year survival rate is about 48% [2]. Although the standard platinum-based chemotherapy and cytoreductive surgery result in complete remission, cancer tends to relapse and disseminate to distant organs in most patients [3].

Recent studies have shown the contribution of the tumor microenvironment (TME) to OC metastasis [4]. Compared to other solid tumors, the TME of epithelial OC is unusual because the cancer cells frequently escape the primary tumor site to create a microenvironment in the peritoneal cavity, known as malignant ascites [5]. Several therapeutic approaches based on angiogenesis, tumor-associated macrophages, cancer-associated fibroblasts, and immune checkpoint blockade are being devised to target the TME [6, 7].

The diversified TME in OC is a manifestation of its high heterogeneity. Jiménez-Sánchez et al. reported both regressing and progressing metastases, with different immune molecular patterns, in the same patient with high-grade serous ovarian cancer (HGSOC) who had undergone multiple chemotherapies [8]. They further reported the co-existence of inflammatory and immune cell-excluded microenvironments in patients with untreated HGSOC, suggesting widespread variation in infiltrating immune cells [9]. Therefore, identifying the subset of highly metastatic cancer cells and OC cell type diversity at the single-cell level is necessary for developing effective clinical biomarkers and treatment strategies for OC.

A TME comprises myeloid-derived suppressor cells (MDSCs), lymphocytes, macrophages, mast cells, neutrophils, and dendritic cells (DCs) and contributes to tumor growth, invasion, and metastasis [10, 11]. Moreover, abundant tumor-associated macrophage (TAM) infiltrates are associated with poor prognoses [12]. The ovarian cancer TME is mostly populated with TAM that exhibits functional plasticity and polarizes into M2-like cells [5, 13]. The immunosuppressive M2-like phenotype induces inflammation, tissue remodeling, and tumor angiogenesis [13].

Identifying prognostic markers of OC using large-scale public gene expression data may provide novel therapeutic targets for OC. We integrated single-cell RNA sequencing (scRNA-seq) and bulk RNA-seq to develop and validate a TAM-based prognostic signature for OC. The TME of HGSOC ascites samples, identified using scRNA-seq data, was highly enriched in M2-like macrophages. *TAP1*, *CD163*, *VSIG4*, *IGKV4-1*, *CD3E*, and *MS4A7* were identified as the major OS-predicting gene signatures using the bulk RNA-seq data. Finally, a prognostic model was validated using a GEO validation dataset.

## RESULTS

### Tumor cell heterogeneity in ovarian cancer

**Figure 1:**
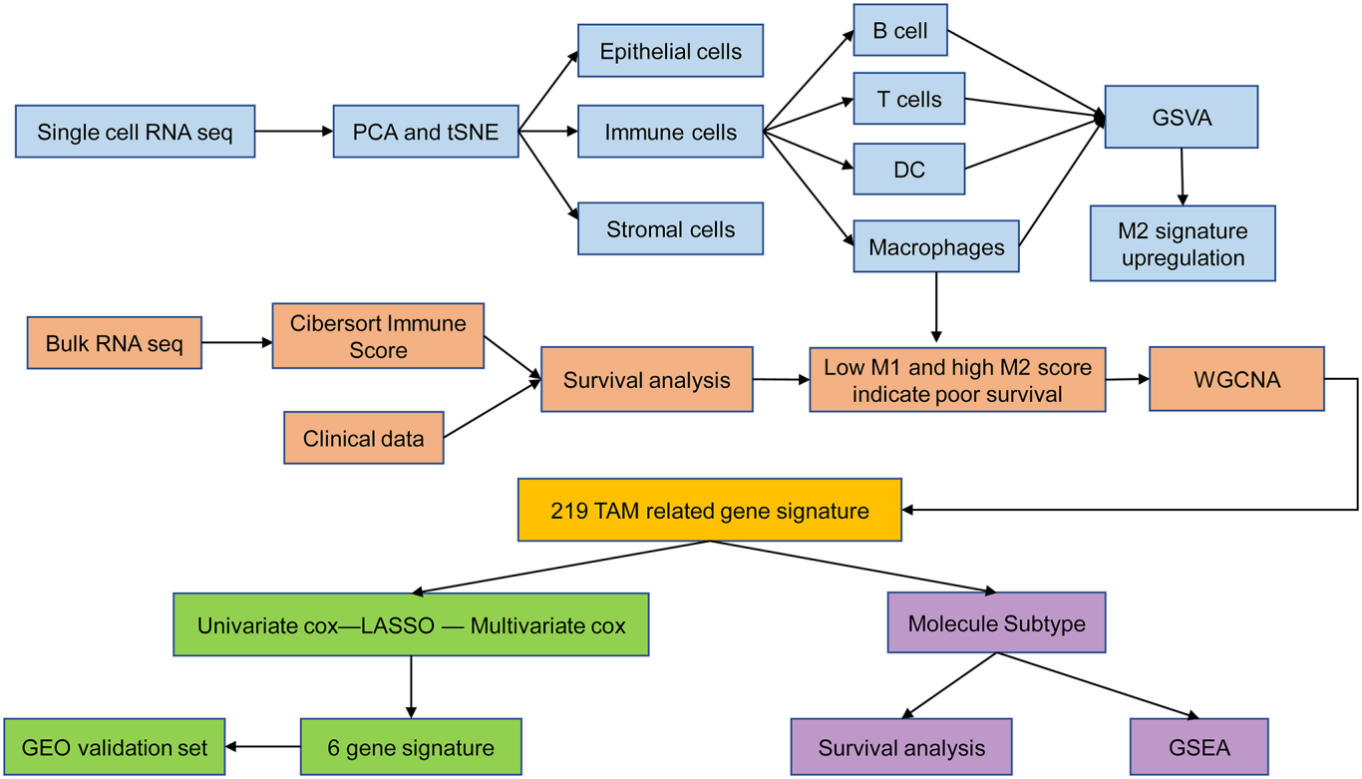
Schematic illustration of the study design.

**Figure 2:**
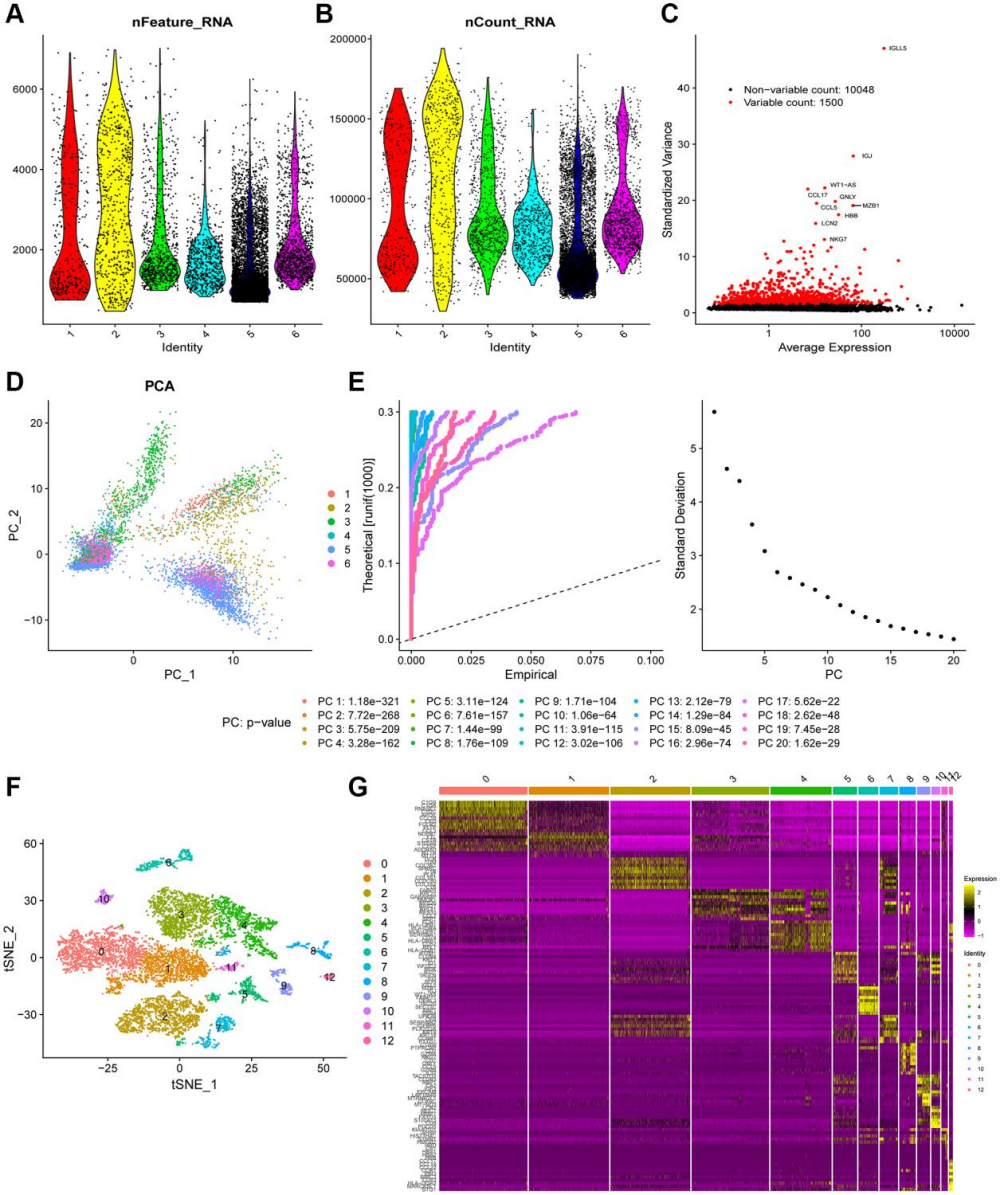
**Heterogeneity in patients with ovarian cancer (OC) was identified using single-cell RNA-seq data.** (**A** and **B**) A total of 9,609 cells from six OC patients were included in the analysis. (**C**) Scatter plots displayed 10,048 corresponding genes in all cells from OC samples. (**D**) The principal component analysis (PCA) revealed unclear separations of OC cells. (**E**) The first 20 principal components with a *p*-value < 0.05 were generated by PCA. (**F**) OC cells were categorized into 13 clusters using the tSNE algorithm with the first 20 principal components. (**G**) The heatmap shows the top 10 differential marker genes of each cluster. A total of 124 unique genes were identified after removing the same marker genes among the clusters.

Figure 1 shows the schematic illustration of the study design. A total of 9,609 cells from ascites samples of patients with OC were obtained for the analysis after quality control (Figure 2A and 2B). Variance analysis showed 1,500 highly variable genes among 10,048 genes. The top 10 genes were identified as *IGLL5*, *IGJ*, *WT1-AS*, *CCL17*, *GNLY*, *CCL5*, *MZB1*, *HBB*, and *NKG7* (Figure 2C). The principal component analysis (PCA) showed a mixed representation of intra-and inter-patient cells (Figure 2D). The *p*-value of the first 20 principal components (PCs) was less than 0.05 (Figure 2E). The highly related genes in the top four PCs are shown in Supplementary Figure 1. Afterward, *t*-distributed stochastic neighbor embedding (tSNE) was performed with PC 1–20 at a resolution of 0.3 to group the OC cells into 13 separate clusters (Figure 2F). A total of 6,453 marker genes were identified and the top 10 differentially expressed genes from the 13 clusters were displayed in the heatmap (Figure 2G).

### Identification of immune cells and GSVA

**Figure 3:**
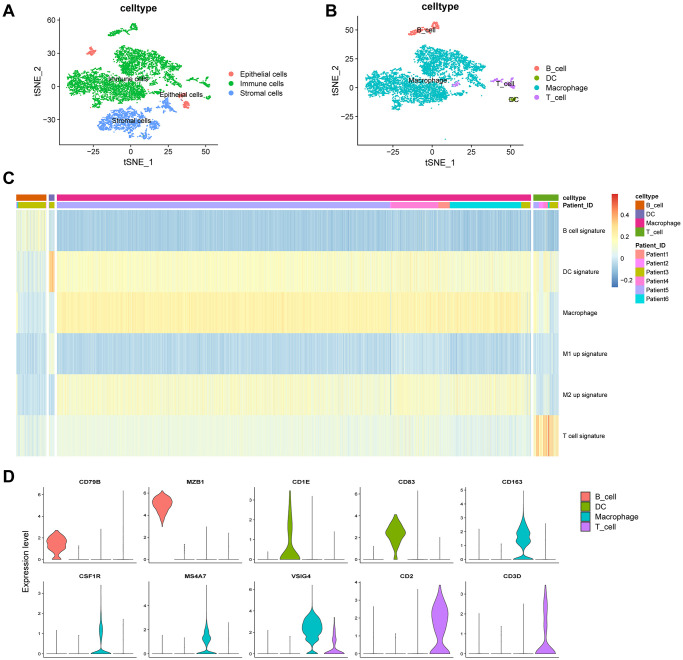
**Clustering of immune cell populations and GSVA enrichment scores.** (**A**) Cell-types visualized using tSNE dimensionality reduction revealed the clustering of tumor-stroma immune cells. (**B**) Immune cells in the tumor microenvironment were annotated into four subpopulations. (**C**) Hierarchical clustering was used using GSVA enrichment scores for gene set for B cell signature, DC signature, macrophage signature, M1 up signature, M2 up signature, and T cell signature. (**D**) Violin plot indicates the genes corresponding to immune cell subpopulations.

We next annotated the cell clusters as epithelial, immune, or stromal (fibroblasts) cells (Figure 3A) (Supplementary Table 1) using previously established cell surface markers. Clusters 9 and 10, containing 419 cells, were classified as epithelial cells; clusters 2 and 7, containing 1,895 cells, were classified as stromal cells; and clusters 0, 1, 3–8, 11, and 12, containing 6,706 cells, were classified as immune cells. We next extracted immune cells (*n* = 6,706) separately and annotated them as B cells, macrophages, dendritic cells (DCs), and T cells (Figure 3B). Clusters 0, 1, 3, and 4, containing 5,930 cells, were classified as macrophages; cluster 6, containing 384 cells, was classified as B cells; cluster 8, containing 320 cells, was annotated as T cells; and cluster 12, containing 72 cells, was classified as DCs.

Gene set variation analysis (GSVA) was performed to analyze the B cell functional status, including cytokine production, naïveness, anti-apoptotic, proliferation, pro-apoptotic functions, and germinal center characteristics. The functional status related to pro-apoptosis showed heterogeneity in the same patient (Supplementary Figure 2). For T cells, we estimated the cytotoxic, native, regulatory, exhausted, co-stimulatory, and G1/S- and G2/M-related gene signatures. The functional status of cytotoxicity in patient 2 was highly enriched (Supplementary Figure 3). Figure 3C shows a comprehensive analysis of the four kinds of immune cells. Macrophages constituted the largest proportion (88.4%) of immune cells. The M2 up signature was highly enriched in macrophages (Figure 3C). Macrophages were marked by CD163, CSF1R, MS4A7, and VSIG4; B cells were marked by CD79B and MZB1; T cells were marked by CD2 and CD3D; and DCs were marked by CD1E and CD83 (Figure 3D). In summary, B cells, T cells, and macrophages showed heterogeneity in the same patient and among different patients.

### High M2 and low M1 phenotypes were correlated with poor survival in ovarian cancer patients

**Figure 4:**
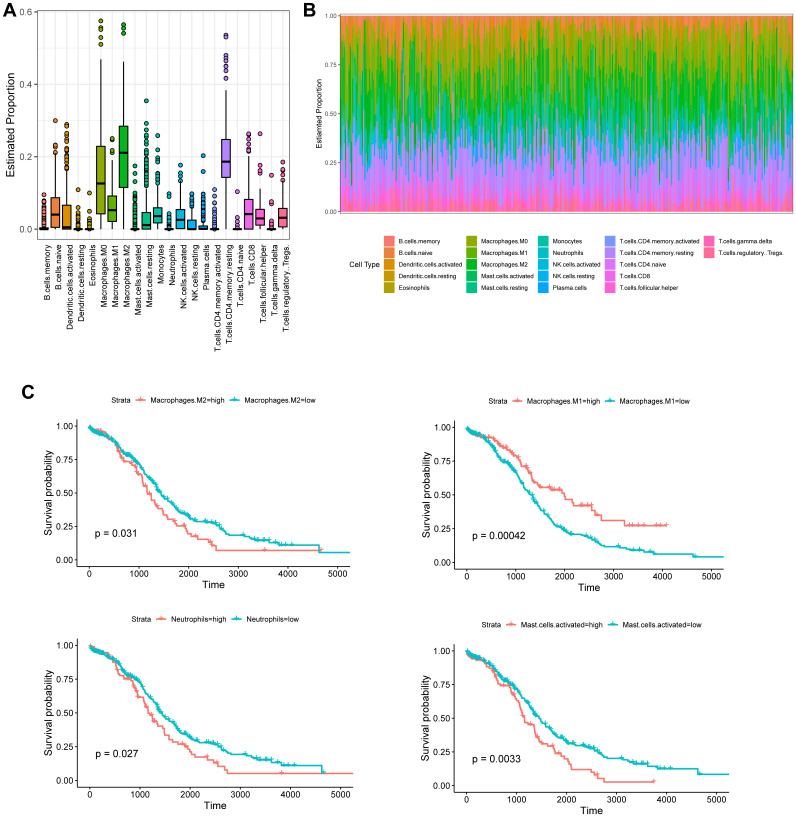
**Tumor-infiltrating immune cell profile of OC samples and survival analysis.** (**A**) Boxplot and barplot (**B**) display the proportion of 22 infiltrating immune cell types in OC samples. (**C**) Kaplan–Meier analysis of overall survival with respect to M2 macrophages, M1 macrophages, activated mast cells, and neutrophils in TCGA-OC.

The CIBERSORT-based Nu support vector regression algorithm was used to evaluate the immune score of the transcribed dataset of bulk RNA-seq obtained from a TCGA-OC cohort. The estimated proportion of 22 immune cell types is shown in Figure 4A and 4B. Among them, four kinds of tumor-infiltrating immune cells were negatively correlated with the OS of OC patients, including M2 macrophages (*p* = 0.031), activated mast cells (*p* = 0.0033), activated memory CD4^+^ T cells (*p* = 0.04), and neutrophils (*p* = 0.027), whereas M1 macrophages (*p* = 0.00042) were positively associated with the OS of OC patients (Figure 4C).

### Identification of TAM-related genes

**Figure 5:**
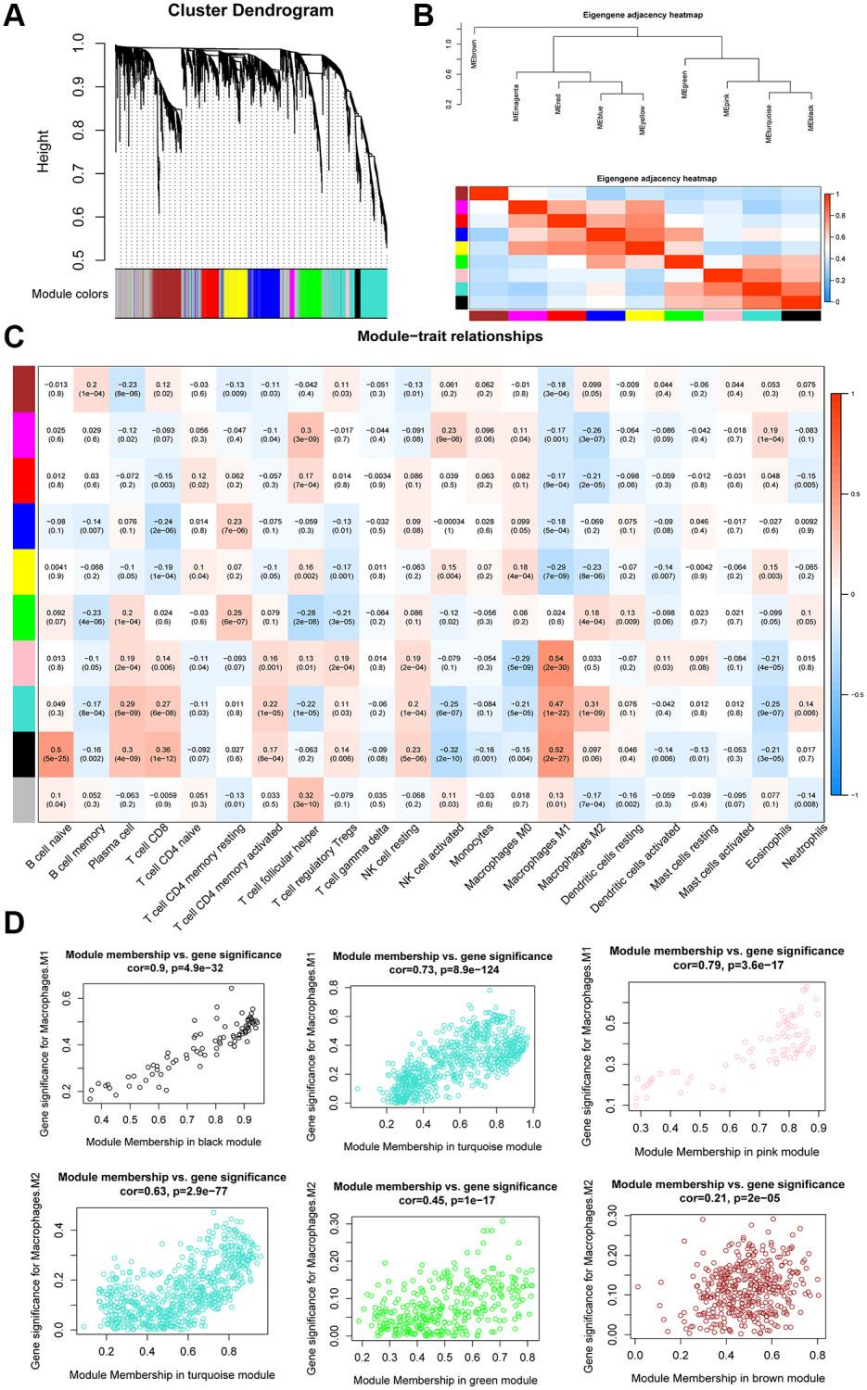
**WGCNA analysis of co-expression modules.** (**A**) Dendrogram of gene modules based on the dynamic hybrid cutting model. Ten modules were constructed. (**B**) Heatmap and hierarchical clustering of adjacencies in module eigengenes. (**C**) Heatmap of the correlation between module eigengenes and the proportion of tumor-infiltrating immune cells. (**D**) Scatter plot of M1 macrophage and M2 macrophage module eigengenes in three modules.

The expression values of 4,043 genes were used to build a gene co-expression network using the WGCNA R package. Pearson’s correlation values and average linkage values were used to cluster the OC samples. A hierarchical clustering tree was constructed using the dynamic hybrid cutting model. Each leaf on the tree represented a single gene. Genes with similar expression patterns were clustered into branches to form a gene module. Furthermore, 10 modules were constructed (Figure 5A), among which the turquoise module exhibited a strong relationship with the black and pink modules (Figure 5B).

We correlated the gene module and CIBERSORT fraction and found that the black module exhibited a high correlation with M1 macrophages (R^2^ = 0.52, *p* = 2e-27) and naïve B cells (R^2^ = 0.5, *p* = 5e-25). The pink module was highly correlated with M1 macrophages (R^2^ = 0.54, *p* = 2e-30) and CD8^+^ T cells (R^2^ = 0.36, *p* = 1e-12), whereas the turquoise module was highly correlated with M1 (R^2^ = 0.47, *p* = 1e-22) and M2 macrophages (R^2^ = 0.31, *p* = 1e-09; Figure 5C). The scatter plots illustrated a strong positive correlation between module membership and gene significance in the black module (cor = 0.9, *p* = 4.9e-32), turquoise module (cor = 0.73, *p* = 8.9e-124), and pink module (cor = 0.79, *p* = 3.6e-17; Figure 5D), indicating that these modules were highly correlated with TAMs. Because we were specifically interested in macrophages, we focused on the black, pink, and turquoise modules that showed a correlation with macrophages; these were identified as TAM-related modules. According to the cut-off criteria, 219 genes in three modules were defined as tumor-associated macrophage-related gene (TAMRG) signatures (Supplementary Table 2).

### Molecular subtypes based on TAMRG signature

**Figure 6:**
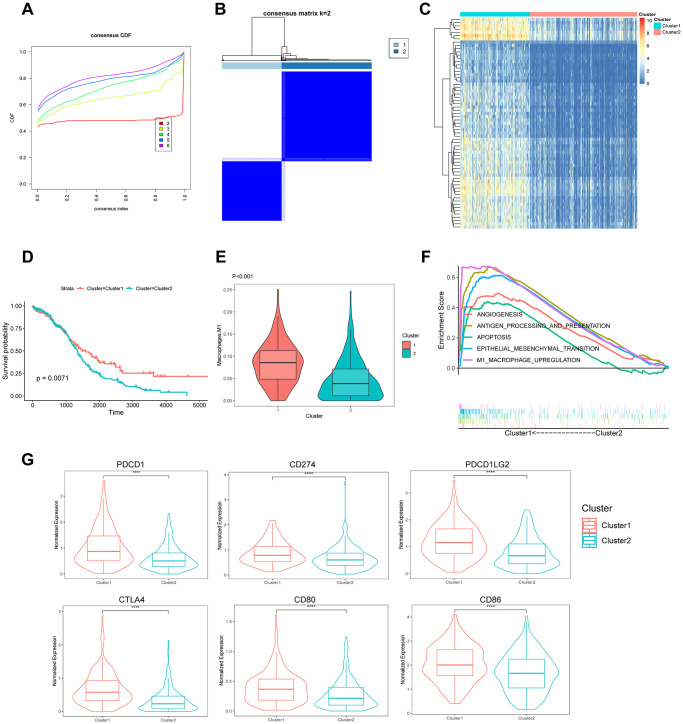
**Identification of the molecular subtypes based on 219 TAMRG signatures.** (**A**) CDF plot of the consensus score (*k* = 2–6). (**B**) Consensus clustering matrix for *k* = 2. (**C**) Heatmap showing differentially expressed genes between the two groups. (**D**) Kaplan–Meier analysis of overall survival for clusters 1 and 2. (**E**) The TAM abundance of two groups is shown in violin plots. (**F**) Upregulated pathways in GSEA. (**G**) The expression of six immune checkpoints between the two molecular subtypes.

The RNA-seq data of 375 patients with OC in the TCGA database were clustered using the unsupervised k-means-based clustering method and the expression patterns of 219 TAMRG signatures. The cumulative distribution function (CDF) plot showed *k* = 2 (2–6) as the optimal number of clusters (Figure 6A). The consensus heatmap divided all OC patients into two clusters: 151 (40.0%) in cluster 1 and 224 (60.0%) in cluster 2 (Figure 6B). The heatmap revealed differentially expressed genes between the two molecular subtypes (Figure 6C). The Kaplan–Meier survival analysis indicated that patients in cluster 2 had worse survival than those in cluster 1 (*p* = 0.0071) (Figure 6D). The violin plot showed that cluster 1 had higher M1 macrophage scores than cluster 2 (*p* = 6.8e-13; Figure 6E). The GSEA revealed the enriched pathways between the two groups. Some of the upregulated pathways included apoptosis, antigen processing and presentation, angiogenesis, epithelial–mesenchymal transition (EMT), and M1 macrophage upregulation in cluster 1 (Figure 6F). We compared six main immune checkpoints between the two molecule subtypes of OC patients. PDCD1 (PD1; *p* = 6.2e-10), CTLA4 (*p* = 6.6e-16), CD274 (PDL1; *p* = 5.4e-06), CD80 (*p* = 7.7e-07), PDCD1LG2 (PDL2; *p* = 1.9e-0), and CD86 (*p* = 1.6e-05) were highly expressed in cluster 1 (Figure 6G).

### Identification of the best TAMRG-based gene signature for predicting survival in OC patients

We identified 25 prognosis-associated TAM-based signatures using univariate Cox analysis in the TCGA training cohort (Supplementary Figure 4A). We identified a 6-gene signature using least absolute shrinkage and selection operator (LASSO) algorithm followed by multivariate Cox analysis (Supplementary Figure 4B and 4C): CD163 (hazard ratio [HR] = 1.19, 95% confidence interval [CI] = 1.05–1.34, *p* < 0.01), transporter 1 (TAP1, HR = 0.74, 95% CI = 0.63–0.87, *p* < 0.001), V-set and immunoglobulin-domain containing 4 (VSIG4, HR = 1.15, 95% CI = 1.04–1.28, *p* < 0.01), immunoglobulin kappa chain variable 4–1 (IGKV4-1, HR = 0.64, 95% CI = 0.47–0.86, *p* < 0.01), CD3E (HR = 0.82, 95% CI = 0.70–0.96, *p* = 0.016), and membrane spanning 4-domains A7 (MS4A7, HR = 1.16, 95% CI = 1.02–1.32, *p* = 0.023). We explored the expression of the 6-gene signature in the scRNA-seq set (Supplementary Figure 5A; MS4A7, VSIG4, and IGKV4-1 were not provided). CD163, MS4A7, and VSIG4 were upregulated in macrophages, whereas CD3E was significantly upregulated in T cells. TAP1 was upregulated in macrophages and B cells. The expression of IGKV4-1 was not detected. Furthermore, Gene Expression Profiling Interactive Analysis (GEPIA) was performed to study the expression of the 6-gene signature in 88 normal (GTEx) samples and 426 OC (TCGA) samples. We noticed that compared with normal samples, CD163 was upregulated, whereas TAP1, CD3E, and IGKV4-1 were downregulated in OC (Supplementary Figure 5B).

### Generation and validation of the 6-gene signature-based prognostic risk score model

**Figure 7:**
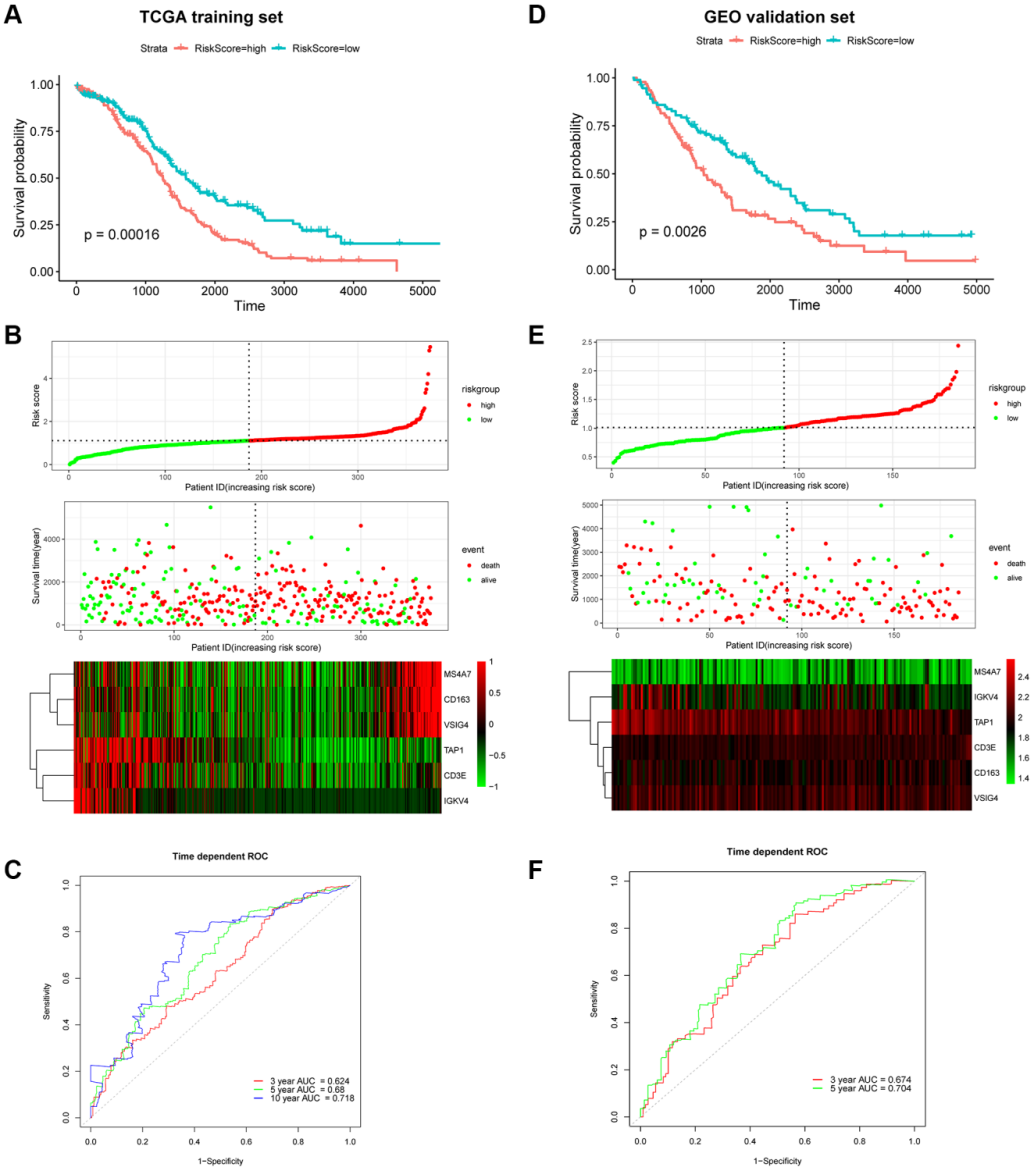
**Prognostic analysis of the 6-gene signature in OC patients.** Kaplan–Meier OS curve of low-risk and high-risk groups in the TCGA training dataset (**A**) and GEO validation dataset (**D**). Risk score distribution in TCGA (**B**) and GEO (**E**) datasets. Upper panel: The curve of risk score. Middle panel: patients’ overall survival status and time. Bottom panel: Heatmaps of gene expression profiles. ROC curve for OS prediction in the TCGA (**C**) and GEO (**F**) datasets.

The risk score was calculated using the following formula: risk score = 0.0284 × ExpCD163 + (–0.0297) × ExpCD3E + (–0.0007) × ExpIGKV4 + (–0.0130) × ExpTAP1 + 0.0009 × ExpVSIG4 + 0.0487 × ExpMS4A7. Patients were dichotomized into low-risk or high-risk groups according to the median risk score (Figure 7B). The Kaplan–Meier survival curve revealed that the OS of the low-risk group was higher than that of the high-risk group (log-rank, *p* = 0.00016; Figure 7A and 7B). The concordance index (C-index) for OS prediction was 0.614 (95% CI = 0.593–0.636). The receiver operating characteristic (ROC) curve demonstrated that areas under the curve (AUC) of 0.624, 0.68, and 0.718 revealed a predictive ability of 3-, 5- and 10-year OS, respectively (Figure 7C). The relationship between the proportion of six infiltrating immune cell types and the risk score was analyzed to validate the effect of the 6-gene signature on TAMRG. The scatter plot showed a highly negative correlation between the risk score and the proportion of infiltrating M1 macrophages (R = 0.38, *p* = 1.4e-14). The proportion of infiltrating M2 macrophages was positively correlated with the risk score (R = 0.29, *p* = 1.3e-08; Supplementary Figure 5C).

Next, the GEO cohort was used to validate the prognostic predictive performance. As shown in Figure 7D and 7E, 185 OC patients were divided into low-risk and high-risk groups. Consistent with the TCGA training cohort, the Kaplan–Meier survival curve suggested that the OS of the high-risk group was considerably lower than that of the low-risk group (log-rank, *p* = 0.0026;). The C-index was 0.611 (95% CI = 0.584–0.638). Moreover, the ROC suggested AUC values of 0.674 and 0.704, indicating that the model could predict 3- and 5-year OS, respectively (Figure 7F).

### Comparison with clinical characteristics and other signatures

We next compared the predictive accuracy of the 6-gene signature with clinical characteristics and published prognostic signatures of OC. Among the 13 survival predictors, TAMRG-based signature exhibited the best mean C-index (0.613) for age (0.593), grade (0.532), stage (0.519), residual (0.556) (Supplementary Table 3), and other signatures (0.516–0.584; Supplementary Table 4). These results revealed that the TAMRG-based signature was an independent prognostic indicator that effectively predicted the prognosis of patients with OC.

## DISCUSSION

Solid tumors are characterized by a unique microenvironment formed by malignant and several non-malignant cells that can modify tumor characteristics [12]. We analyzed the heterogeneity of tumor-infiltrating immune cells in OC using scRNA-seq and bulk RNA-seq data. At the single-cell level, the majority of non-malignant cells were immune cells, identified as four distinct clusters of B cells, T cells, DCs, and macrophages. Both inter-and intra-patient heterogeneity was observed for tumor-infiltrating immune cells in HGSOC. Macrophages and T cells exhibited immunosuppressive characteristics: macrophages with an M2 phenotype and T cells with an exhausted phenotype. M2 macrophages express the ligand receptors for PD-1 and CTLA-4, whose activation inhibits T cell proliferation and cytotoxic function, thus contributing to the formation of an immunosuppressive TME [14, 15]. These findings suggested that a distinct immune system status and dynamic immune cell interactions in the surrounding microenvironment contributed to the wide tumor heterogeneity of ovarian cancer transcriptome.

The bulk RNA-seq data were analyzed using CIBERSORT to estimate tumor-infiltrating immune cell subsets and their correlation with prognosis. Activated mast cells, neutrophils, M2 macrophages, and activated memory CD4^+^ T cells were negatively correlated with OS of OC patients, whereas M1 macrophage infiltration indicated better clinical outcomes. Similarly, CD4^+^CD25^+^FOXP3^+^ Treg cell infiltration was associated with high mortality and decreased survival in 104 individuals affected with OC [16]. Although the increased density of M2-like TAM is known to be associated with poor OS [17–19], the relationship between M2 density and OS in OC remains controversial. For instance, Zhang et al. [20] reported no correlation between these two factors, whereas Lan et al. [21] found a negative correlation, consistent with our results. These differences in the findings could be ascribed to varying types of tumor tissues.

A total of 219 TAMRG signatures were established by WGCNA. OC patients with M1-like TAM reported a better prognosis, suggesting that TAMRG-based patient classification could effectively predict patient survival. Although GSEA revealed that M1-like TAM was enriched in immune-related, angiogenesis, EMT, and JAK-STAT3 pathways, M2 macrophages constituted the dominant population in the TAM of OC and were implicated in tumor angiogenesis, invasion, metastasis, and early recurrence [12, 22, 23]. The simple dichotomy of M1/M2 macrophages is insufficient to explain the complexity of TAM heterogeneity [24]. Classically, M1/M2 phenotypes are extremes of a continuum of activation states [24, 25], whereas the TAM subset shares characteristics of both M1 and M2 phenotypes [26]. For example, a recent study revealed that TAMs simultaneously express M1/M2 markers, with early-stage TAMs co-expressing T cell co-stimulatory and co-inhibitory receptors [27]. Similarly, Müller identified phenotypic differences in TAMs from distinct lineages at single-cell resolution in human gliomas. These results indicate that TAMs frequently co-express M1/M2 markers in single cells, making it difficult to isolate M1 and M2 phenotypes [28]. Thus, TAMs exhibit functional plasticity and intermediate states, resulting in reversible changes in their distribution and functional states under different microenvironment stimuli [24, 29–31].

Immunotherapy has emerged as a promising treatment strategy for cancer that alleviates the immunosuppression status of the tumor. For instance, an anti-PD-1 antibody is known to prolong the progression-free survival and OS of patients with melanoma [32] and non-small cell lung cancer [33]. Unfortunately, clinical trials involving immune checkpoint inhibitors either as a single agent or in combination with other therapeutic modalities have been unsuccessful in OC. Our results revealed that cluster 1 with an increased macrophage M1 density expressed high levels of PD1/PDL1/PDL2 and CTLA4/CD80/CD86 molecules, indicating that patients in cluster 1 may benefit more from anti-PD1 and anti-CTLA4 therapies than those in cluster 1.

A novel 6-gene signature risk score module was successfully established and validated using a GEO dataset. Of the six genes, a high expression of *CD163*, *VSIG4*, and *MS4A7* was related to poor prognosis, whereas that of *CD3E*, *IGKV4*, and *TAP1* was associated with a favorable prognosis. Some of these genes have been implicated in cancers. For example, CD163(+) TAMs correlate with poor prognosis, OS, and metastasis of various malignancies. Chen et al. reported that CD163 contributes to gliomagenesis via CK2, and its high expression is associated with an unfavorable patient prognosis [34]. Similarly, CD163(+) TAMs were associated with poor OS and increased microvessel density in gastric cancer [35]. *VSIG4* is overexpressed in OC [36]. Agnes et al. demonstrated that underexpressed *TAP1* resulted in low infiltration of macrophages and poor prognosis in patients with colorectal cancer [37]. Univariate and multivariate Cox regression analyses revealed that the 6-gene signature could be applied as an independent prognostic factor. We believe this prognostic module is the first to incorporate a TAM-related signature to predict survival in patients with OC. Although the absolute value of the prognostic module C-index was low, it was superior to the traditional clinical characteristics. Therefore, it can be used to estimate the prognosis of patients and classify them into distinct subgroups for effective treatment.

Our study had certain limitations. The data of a few patients acquired from TCGA and GEO databases were incomplete in terms of grade and medical history (e.g., unavailable for multivariate Cox analysis). In addition, this was a retrospective study; further prospective, large-scale trials are warranted to verify its clinical application.

## CONCLUSIONS

We constructed a novel TAMRG-based, the 6-gene prognostic signature for patients with OC. The TAMRG-based signature could serve as a potential target for the prognosis and predicting the therapeutic response in patients with OC.

## MATERIALS AND METHODS

### Dataset collection and preprocessing

We used both the bulk RNA-seq data and scRNA-seq data of human OC samples. The scRNA-seq data (GSE146026) consisted of 9,609 cells from six ascites samples of HGSOC and were acquired from the GEO database. The single-cell library was constructed on a 10× genomics platform and read on an Illumina NextSeq 500 sequencing system. The bulk RNA-seq data of patients with OC were downloaded from the GEO and TCGA databases. We included 375 OC samples with available clinical characteristics from the TCGA database as the training dataset and 185 OC samples from the GEO database (GSE26712) as the validation dataset.

### Single-cell RNA-seq analysis

The R4.0.5 software was used to perform all analyses. The Seurat 3.0 package was used for scRNA-seq data quality control, filtering, statistical analysis, and subsequent analysis [38]. First, cells with < 200 detected genes, genes detected in < 3 cells, and mitochondrial genes ≥ 5% were used as the filtering criteria. In total, 9,609 cells were included in the study. Next, a linear regression model was used to normalize the gene expression. Data were analyzed using PCA to visualize the available dimensions (*p*-value < 0.05). The first 20 PCs were used for tSNE with a resolution of 0.3 for dimension reduction and clustering. The differentially expressed genes were identified using the Wilcoxon test and FindAllMarkers function of Seurat. Marker genes were identified using the following cut-off criteria: |log2[fold change (FC)]| > 0.5 and *p*-value < 0.05. Afterward, three cell types were annotated using the established cell surface marker genes [39]. To further characterize the immune cells, we annotated the cells using the singleR package and corrected with the CellMarker database. The list of cell surface markers is shown in Supplementary Table 1.

### Gene set variation analysis of immune cells

GSVA is an unsupervised gene set enrichment method to estimate the variations in pathway activity within a sample population [40]. The enrichment scores of the gene sets were evaluated using the GSVA package in R. Immune cell-related gene sets were derived from supplementary documents provided by Chung et al. [41].

### Estimate of the abundance of tumor immune infiltration and survival analysis

The CIBERSORT R script was used to calculate the abundance of 22 tumor-infiltrating immune cell types in the TCGA OC cohort based on the bulk RNA-seq dataset [42]. The median of each immune cell enrichment score was used to separate the samples into two groups to compare the OS. The R package “survival” and “survminer” were used for computing the survival.

### Construction of weighted correlation network analysis

WGCNA analysis was implemented using the R package “WGCNA” [43]. To construct a signed, scale-free co-expression gene network, the scale-free topology fitting index R^2^ > 0.85 and power of β = 3 were selected as soft-threshold parameters. We used the dynamic hybrid cutting method to classify genes with similar expression patterns; the minimum size cut-off of the module was 30. Module eigengenes were used to perform the component analysis of each module. We estimated the correlation between tumor-infiltrating immune cell enrichment score and module eigengenes to determine the significance of modules using Pearson’s test. The selected macrophage subtypes and modules were used for follow-up analysis. The major genes were determined by calculating the gene significance (GS) and module membership (MM). The genes in the module with |GS| > 0.2 and MM > 0.8 were considered significant.

### Identification of molecular subtypes

The R package “ConsensusClusterPlus” was used to perform k-means-based unsupervised consensus clustering based on the expression patterns of TAM-related gene signature [44]. We obtained consensus matrices and cumulative distribution function (CDF) plots with a set of parameters, including 1,000 iterations and an 80% resampling rate in Pearson’s correlation. Next, we compared OS between different clusters using the Kaplan–Meier survival analysis. GSEA was used to explore the potential molecular mechanisms. In addition, we compared six immune checkpoints (PDCD1, CTLA4, CD274, CD80, PDCD1LG2, and CD86) between different clusters.

### Establishment and estimation of the prognostic risk score model

First, the univariate Cox regression analysis was used to assess the associations between TAM-related genes and survival in the TCGA training set. Second, the LASSO algorithm and multivariate Cox regression analysis were applied to identify prognosis-related genes with *p* < 0.05. Subsequently, a prognostic risk model was established based on the major prognosis-related TAMRG-based genes. The risk score was calculated by a linear method to assemble the Cox coefficient and prognostic gene expression using the following formula: Risk score = β1 × Expgene1 + β2 × Expgene2 + … + βn × Expgenen, where “β” and “Exp” represent the regression coefficient and the expression of prognostic genes, respectively.

The patients in the TCGA training set were divided into high-risk and low-risk groups according to the median risk score. Kaplan–Meier survival analysis was used to compare the OS of these two groups. Time-dependent ROC curve analysis and Harrell’s C-index were applied using the “survivalROC” and “survminer” packages in R to evaluate the prediction accuracy of the prognostic risk model. Finally, a validation cohort was obtained from the GEO database to validate the prognostic value of the risk model. In addition, we performed a comparison of the C-index between the prognostic model and age, stage, grade, residual, and eight published signatures.

### Statistical analyses

Student’s *t*-test was used to compare the mean values between the two groups. Cox proportional hazard models were applied to assess the associations between factors and OS. Survival curves were drawn using the Kaplan–Meier method and compared using log-rank tests. The C-index and the ROC curve were estimated using survival, survminer, and survivalROC packages in the R software.

Statistical analyses were performed using the R version 4.0.5 (The R Foundation). The *p*-values were two-tailed, and *p* < 0.05 was considered significant.

### Availability of data and materials

The public datasets used in our study are available at https://portal.gdc.cancer.gov/ and https://www.ncbi.nlm.nih.gov/.

## Supplementary Materials

Supplementary Figures

Supplementary Tables
