# Supplementary material for: Integrated analysis of tumor-associated macrophage infiltration and prognosis in ovarian cancer
Source: Aging (Albany NY). 2021 Oct 11;13(19):23210–32. doi: 10.18632/aging.203613 (PMC8544311; doi:10.18632/aging.203613)
Supplement: Supplementary Figures [file aging-13-203613-s001.pdf]

SUPPLEMENTARY FIGURES

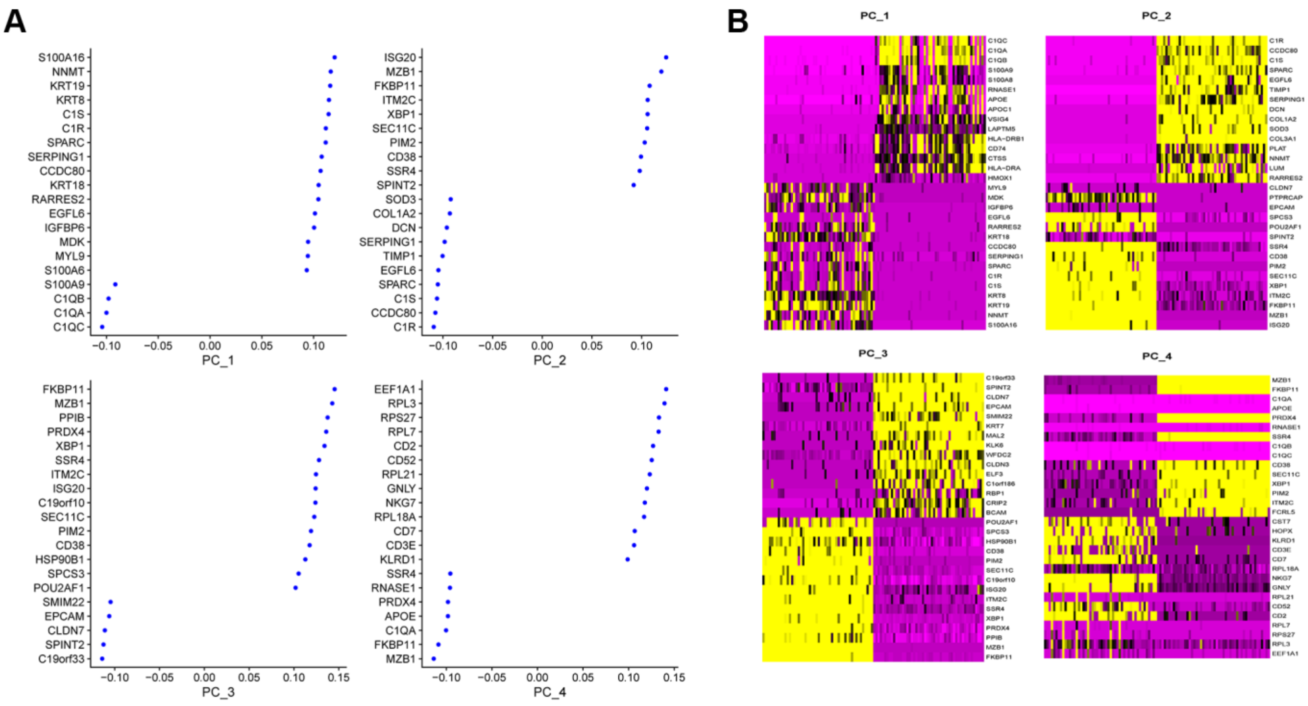

Supplementary Figure 1. Dot plots (**A**) and heatmaps (**B**) display the top 20 highly correlated genes in PC1 to PC4.

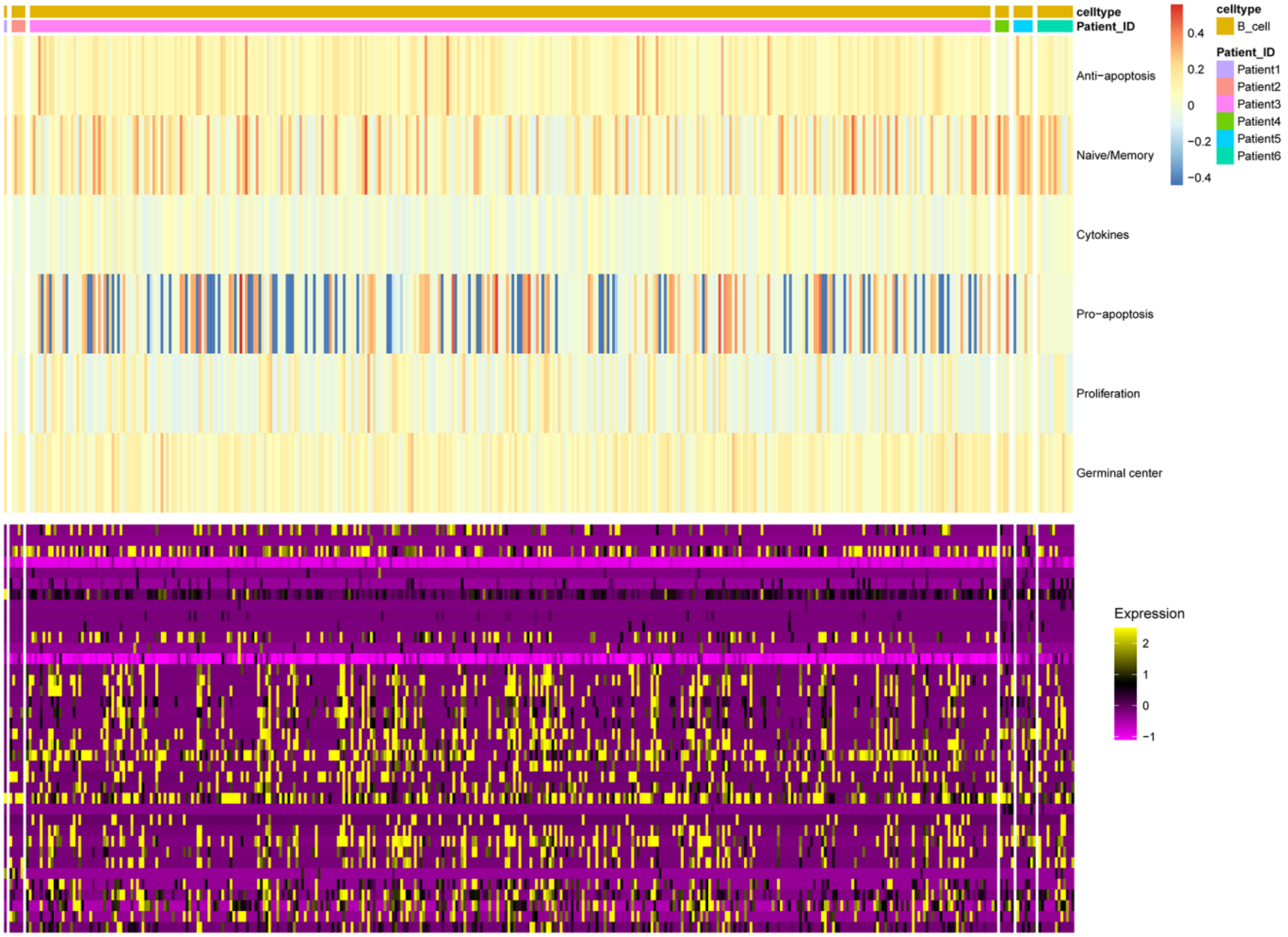

Supplementary Figure 2. B-cell signatures in OC samples. Upper panel: GSVA enrichment scores of B cells in functional status. Bottom panel: heatmap of genes in the above gene sets.

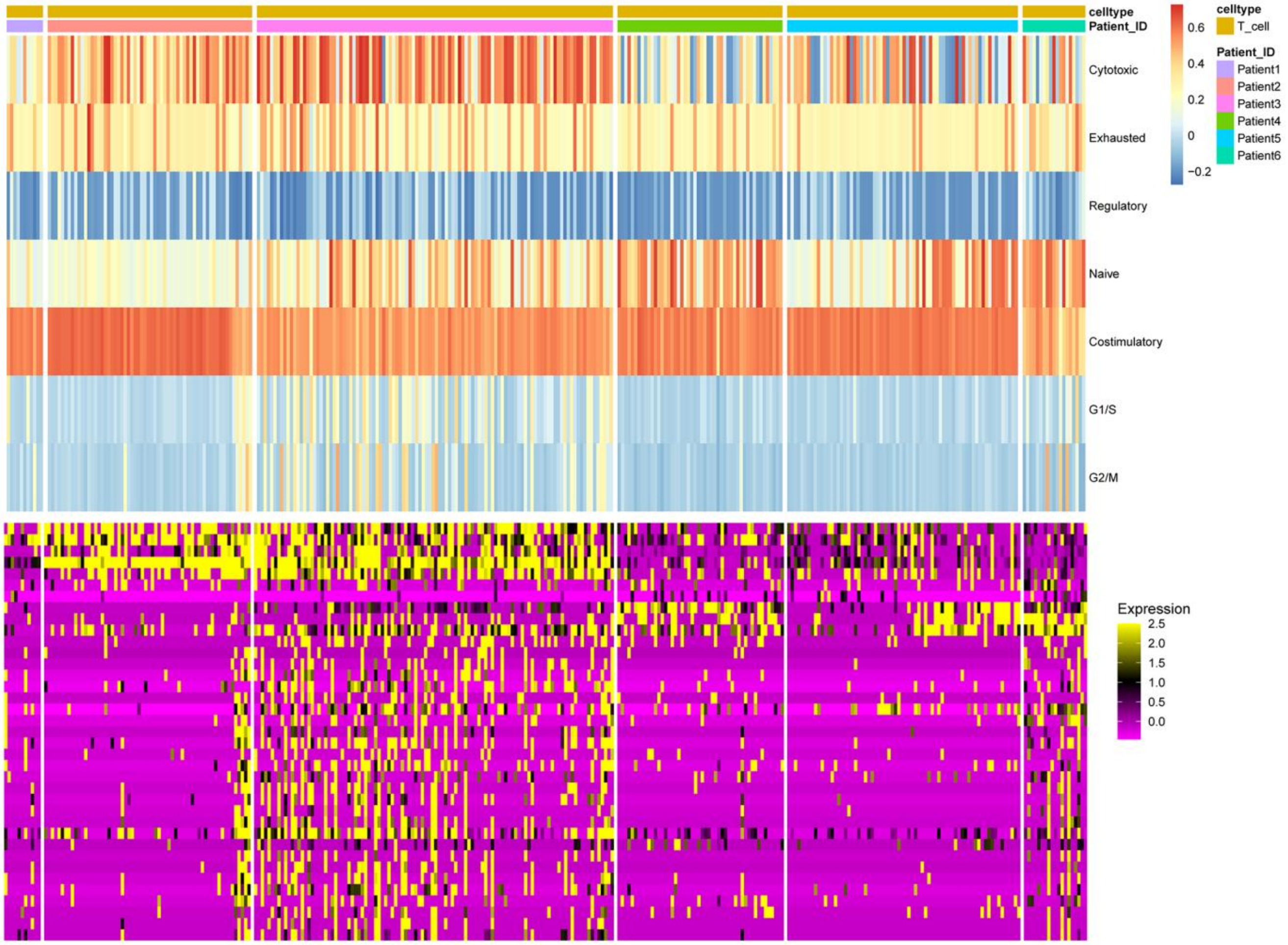

Supplementary Figure 3. T-cell signatures in OC samples. Upper panel: GSVA enrichment scores of T cells in functional status. Bottom panel: heatmap of genes in the above gene sets.

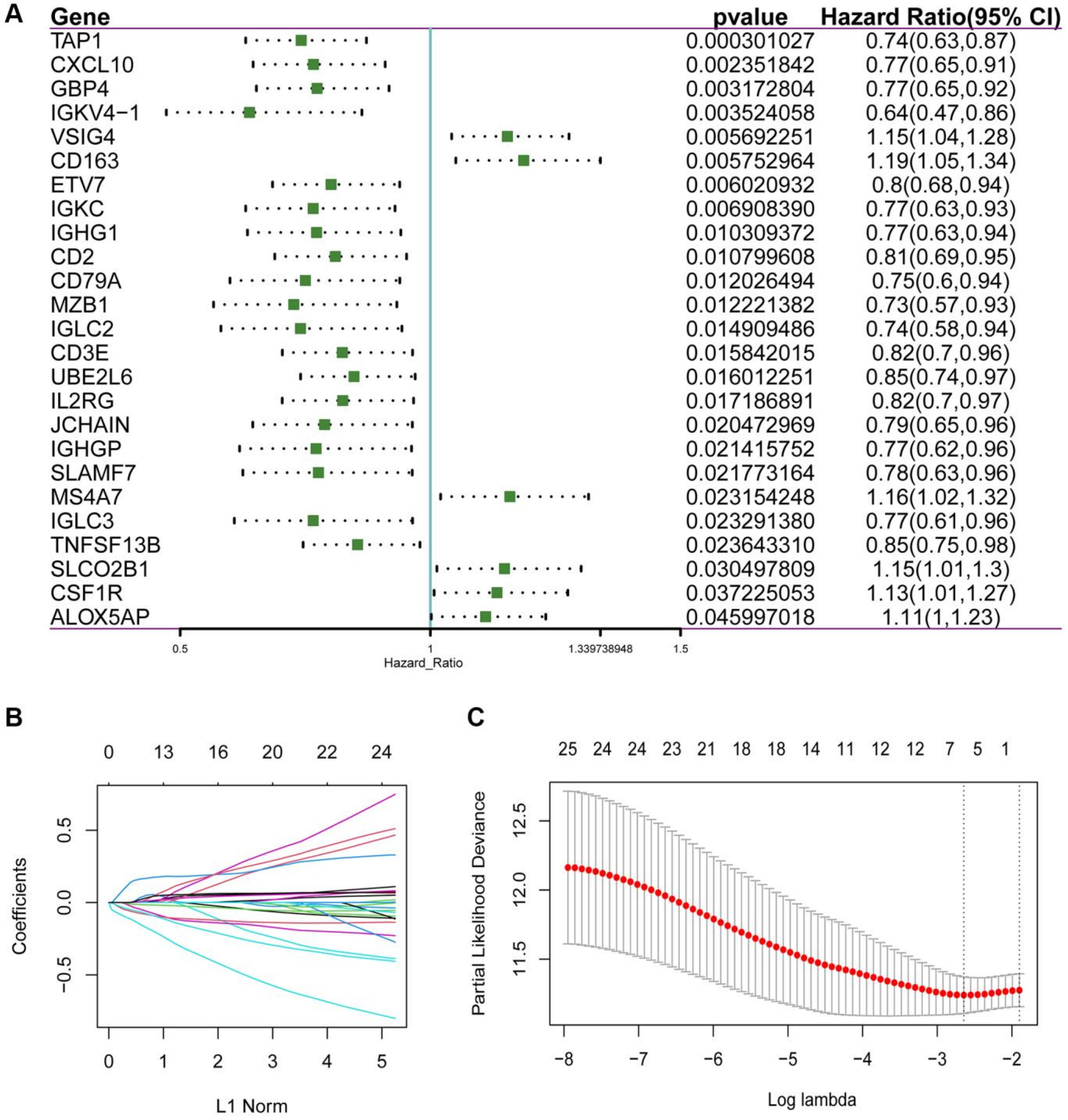

Supplementary Figure 4. (**A**) Univariate Cox regression analysis of 25 prognostic genes from 219 TAMRG. (**B**) LASSO coefficient profiles of 25 genes. (**C**) LASSO regression with 10-fold cross-validation resulted in six prognostic genes using an optimal lambda value.

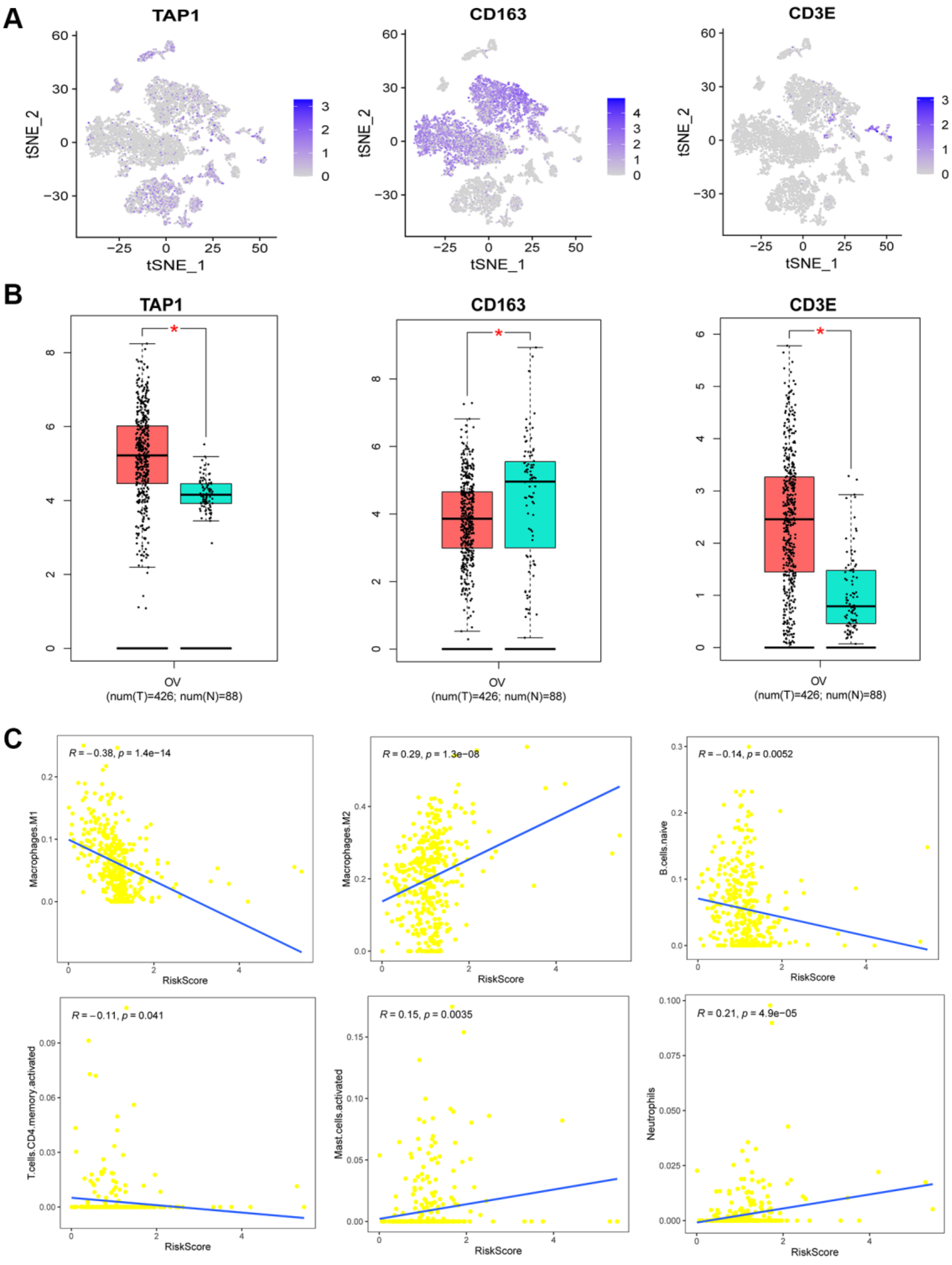

Supplementary Figure 5. (**A**) The expression of *TAP1*, *CD163*, and *CD3E* in scRNA-seq set. (**B**) The expression of *TAP1*, *CD163*, and *CD3E* in 426 OV (TCGA) samples and 88 normal (GTEx) samples. (**C**) The relationship between the proportion of six infiltrating immune cell types and the risk score.
