# Supplementary material for: Integrated analysis of tumor-associated macrophage infiltration and prognosis in ovarian cancer
Source: Aging (Albany NY). 2021 Oct 11;13(19):23210–32. doi: 10.18632/aging.203613 (PMC8544311; doi:10.18632/aging.203613)
Supplement: Supplementary Tables [file aging-13-203613-s002.pdf]

## SUPPLEMENTARY TABLES

**Supplementary Table 1. The list of cell surface markers used to identify cell cluster.**

| Cells | Markers |
|---|---|
| Epithelial | PECAM1, CD34, VWF, EPCAM, SFN, KRT19 |
| Fibroblasts | ACTA2, MCAM, MYLK, MYL9, FAP, THY1 |
| Immune cells | PTPRC, CD3G, CD3E, CD79A, BLNK, CD68, CSF1R, MARCO, CD207 |
| B cell | CD79A, CD79B, CD19, MS4A1, MZB1 |
| T cell | CD3D, CD3E, CD3G, CD2 |
| Macrophages | HLA-DMB, HLA-DQA1, HLA-DRB5, CD68, AIF1, CD74, CYBB, DOK3, KYNU, METRNL, MS4A6A, MS4A7, PIM1, PLAC8, PLBD1, PLTP, S100A11, SH3BGRL, SPINT2, SYNGR2, TREM1, CCL2, CD93, CIB1, CREM, SAMHD1, TMEM123, DSE, FGR, FOSL2 |
| | FPR3, GPR65, ITGA4, PTPN7, SAMHD1, TGFBI, THBD |
| | VOPP1 |
| Dendritic cell (DC) | HLA-DPB1, HLA-DQB1, HLA-DRA, HLA-DPA1, CD1E, CD83 |

**Supplementary Table 2. 219 TAMRG signature.**

| Gene symbol |
|---|
| ABI3, ADAP2, AIF1, ALOX5AP, AOAH, APBB1IP, APOBR, APOL6, ARHGAP30, ARHGAP9, ARHGDIB, BIN2, BTK, C1orf162, C1QA, C1QB, C1QC, C3AR1, CCR1, CCR5, CD14, CD163, CD180, CD2, CD300A, CD37, CD3E, CD4, CD48, CD53, CD79A, CD84, CD86, CLEC7A, CMPK2, CSF1R, CSF2RA, CSF2RB, CTSS, CXCL10, CYBB, CYTH4, CYTIP, DDX60, DOCK2, DOK2, DOK3, ETV7, EVI2A, EVI2B, FAM78A, FCER1G, FCGR1A, FCGR2A, FCGR2B, FCGR3A, FERMT3, FGL2, FPR3, FYB1, GBP4, GIMAP4, GIMAP6, GNA15, GPR65, GPR84, HAVCR2, HCK, IFI35, IFI44, IFI44L, IFIH1, IFIT2, IFIT3, IGHA1, IGHG1, IGHG2, IGHG3, IGHGP, IGHM, IGHV1-18, IGHV1-2, IGHV1-46, IGHV1-69, IGHV1-69D, IGHV3-11, IGHV3-15, IGHV3-21, IGHV3-23, IGHV3-30, IGHV3-33, IGHV3-48, IGHV3-49, IGHV3-53, IGHV3-66, IGHV3-7, IGHV3-74, IGHV4-28, IGHV4-31, IGHV4-34, IGHV4-39, IGHV4-59, IGHV4-61, IGHV5-51, IGKC, IGKV1-12, IGKV1-16, IGKV1-17, IGKV1-27, IGKV1-39, IGKV1-5, IGKV1-6, IGKV1-8, IGKV1-9, IGKV3-11, IGKV3-15, IGKV3-20, IGKV3D-20, IGKV4-1, IGLC2, IGLC3, IGLL5, IGLV1-40, IGLV1-44, IGLV1-47, IGLV1-51, IGLV2-11, IGLV2-14, IGLV2-23, IGLV2-8, IGLV3-1, IGLV3-19, IGLV3-21, IGLV3-25, IGLV4-69, IGLV6-57, IGSF6, IKZF1, IL10RA, IL2RB, IL2RG, ITGAL, ITGAM, ITGAX, ITGB2, JCHAIN, LAIR1, LAPTM5, LAT2, LCP2, LILRB1, LILRB2, LILRB4, LPXN, LRRC25, LSP1, LY86, MNDA, MPEG1, MPP1, MS4A4A, MS4A6A, MS4A7, MSR1, MX1, MYO1F, MZB1, NCF1, NCF2, NCF4, NCKAP1L, NFAM1, NPL, OAS1, OAS2, OAS3, OASL, OSCAR, PARP12, PARP14, PARP9, PCED1B-AS1, PDCD1LG2, PLEK, PLSCR1, PTPRC, RAC2, RASAL3, RCSD1, RNASE6, RSAD2, SAMD9, SAMD9L, SAMSN1, SASH3, SELPLG, SLA, SLAMF7, SLAMF8, SLC37A2, SLCO2B1, SP100, SPI1, SRGN, STX11, TAGAP, TAP1, TBXAS1, TLR2, TLR7, TNFAIP8L2, TNFSF13B, TRIM22, TRPV2, TYMP, TYROBP, UBE2L6, VSIG4,WAS |

**Supplementary Table 3. Univariate and multivariate Cox proportional hazards analyses of risk score and clinicopathological variables in the TCGA training set.**

| Variable | TCGA training set ($n$ = 375) | | | |
|---|---|---|---|---|
| | Univariate analysis | | Multivariate analysis | |
| | HR (95% CI) | $P$ value | HR (95% CI) | $P$ value |
| Age | 1.02 (1.008–1.032) | 0.001 | 1.017 (1.005–1.030) | 0.0059 |
| Stage | 2.115 (0.938–4.766) | 0.04 | 1.71 (0.755–3.874) | 0.198 |
| Grade | 1.183 (0.820–1.706) | 0.4 | – | – |
| M(M1/M0/Mx) | 1.262 (0.90–1.77) | 0.18 | – | – |
| N(N1/N0/Nx) | 1.428 (0.836–2.439) | 0.19 | – | – |
| Residual | 1.609 (1.210–2.139) | 0.002 | 1.464 (1.095–1.958) | 0.01 |
| Risk score | 1.604 (1.38–1.865) | 3e-07 | 1.596 (1.366–1.865) | 4.07e-09 |

Abbreviations: HR: hazard ratio; CI: confidence interval; M: metastasis; N: lymph node.

**Supplementary Table 4. C-index (standard error) of the prognostic signatures and clinical characteristics in the nine datasets.**

| Study | Training set | Validation set (average) | Overall |
|---|---|---|---|
| Our study | 0.614 (0.022) | 0.611 (0.028) | 0.613 |
| Crijns 2009 [45] | 0.54 (0.021) | 0.560 (0.023) | 0.55 |
| Denkert 2009 [46] | 0.566 (0.021) | 0.541 (0.023) | 0.554 |
| Hernandez 2010 [47] | 0.526 (0.021) | 0.510 (0.023) | 0.518 |
| Kang 2012 [48] | 0.542 (0.021) | 0.514 (0.023) | 0.528 |
| Kernagis 2012 [49] | 0.54 (0.021) | 0.561 (0.023) | 0.55 |
| Konstantinopoulos 2010 [50] | 0.495 (0.021) | 0.529 (0.023) | 0.512 |
| Sabatier 2011 [51] | 0.555 (0.021) | 0.543 (0.023) | 0.549 |
| Yoshihara 2012 [52] | 0.592 (0.021) | 0.576 (0.023) | 0.584 |

## REFERENCES


45. Crijns AP, Fehrmann RS, de Jong S, Gerbens F, Meersma GJ, Klip HG, Hollema H, Hofstra RM, te Meerman GJ, de Vries EG, van der Zee AG. Survival-related profile, pathways, and transcription factors in ovarian cancer. PLoS Med. 2009; 6:e24. https://doi.org/10.1371/journal.pmed.1000024 PMID:19192944

46. Denkert C, Budczies J, Darb-Esfahani S, Györffy B, Sehouli J, Könsgen D, Zeillinger R, Weichert W, Noske A, Buckendahl AC, Müller BM, Dietel M, Lage H. A prognostic gene expression index in ovarian cancer - validation across different independent data sets. J Pathol. 2009; 218:273–80. https://doi.org/10.1002/path.2547 PMID:19294737

47. Hernandez L, Hsu SC, Davidson B, Birrer MJ, Kohn EC, Annunziata CM. Activation of NF-kappaB signaling by inhibitor of NF-kappaB kinase beta increases aggressiveness of ovarian cancer. Cancer Res. 2010; 70:4005–14. https://doi.org/10.1158/0008-5472.CAN-09-3912 PMID:20424119

48. Kang J, D'Andrea AD, Kozono D. A DNA repair pathway-focused score for prediction of outcomes in ovarian cancer treated with platinum-based chemotherapy. J Natl Cancer Inst. 2012; 104:670–81. https://doi.org/10.1093/jnci/djs177 PMID:22505474

49. Kernagis DN, Hall AH, Datto MB. Genes with bimodal expression are robust diagnostic targets that define distinct subtypes of epithelial ovarian cancer with different overall survival. J Mol Diagn. 2012; 14:214–22. https://doi.org/10.1016/j.jmoldx.2012.01.007 PMID:22490445

50. Konstantinopoulos PA, Spentzos D, Karlan BY, Taniguchi T, Fountzilas E, Francoeur N, Levine DA, Cannistra SA. Gene expression profile of BRCAness

that correlates with responsiveness to chemotherapy and with outcome in patients with epithelial ovarian cancer. J Clin Oncol. 2010; 28:3555–61. https://doi.org/10.1200/JCO.2009.27.5719 PMID:20547991

51. Sabatier R, Finetti P, Bonensea J, Jacquemier J, Adelaide J, Lambaudie E, Viens P, Birnbaum D, Bertucci F. A seven-gene prognostic model for platinum-treated ovarian carcinomas. Br J Cancer. 2011; 105:304–11. https://doi.org/10.1038/bjc.2011.219 PMID:21654678

52. Yoshihara K, Tsunoda T, Shigemizu D, Fujiwara H, Hatae M, Fujiwara H, Masuzaki H, Katabuchi H, Kawakami Y, Okamoto A, Nogawa T, Matsumura N, Udagawa Y, et al, and Japanese Serous Ovarian Cancer Study Group. High-risk ovarian cancer based on 126-gene expression signature is uniquely characterized by downregulation of antigen presentation pathway. Clin Cancer Res. 2012; 18:1374–85. https://doi.org/10.1158/1078-0432.CCR-11-2725 PMID:22241791
